# The Empowering Role of Web-Based Help Seeking on Depressive Symptoms: Systematic Review and Meta-analysis

**DOI:** 10.2196/36964

**Published:** 2023-02-02

**Authors:** Nicole Bizzotto, Laura Marciano, Gert-Jan de Bruijn, Peter Johannes Schulz

**Affiliations:** 1 Università della Svizzera italiana Lugano Switzerland; 2 Harvard T H Chan School of Public Health and Dana-Farber Cancer Institute Boston, MA United States; 3 Department of Communication Studies Universiteit Antwerpen Antwerpen Belgium; 4 Department of Communication & Media Ewha Womans University Seoul Republic of Korea

**Keywords:** web-based help-seeking, support groups, depressive symptoms, internet, mental health, empowerment

## Abstract

**Background:**

Most research on web-based help seeking for mental health problems has focused on the antecedents of this behavior. Therefore, little is known about the outcomes of web-based help seeking in general or in specific mental health issues.

**Objective:**

This study was a systematic review and meta-analysis of the literature on the antecedents and consequences of web-based help-seeking behaviors for depressive symptoms.

**Methods:**

A systematic literature search was carried out in 6 scientific databases, leading to 48 studies (for a total of 314,921 participants) included in the qualitative synthesis and 19 included in the meta-analysis.

**Results:**

The results indicated a positive relationship between depressive symptoms and web-based help-seeking behaviors through online support groups (*r*=0.089; *P*=.009), and Generation Z (*r*=0.102; *P*=.008) tended to participate in support groups more than previous generations. In addition, web-based help seeking was positively related to empowerment (*r*=0.245; *P*=.004). Other forms of support reported included the internet and specific self-help tools, but no significant relationships were found with depressive symptoms.

**Conclusions:**

More studies examining the outcomes are needed, together with a more rigorous assessment of web-based help-seeking behaviors. Ultimately, we propose a summary framework for the literature on this topic, including the antecedents, patterns of use, and outcomes of web-based help seeking in the context of depressive symptoms.

## Introduction

### Background

According to the World Health Organization [1], more than 200 million people worldwide report having depressive symptoms annually. However, despite the availability of different treatments, the treatment gap (ie, the difference between the number of people who need care and those who receive care) is 72%-93% [2]. Reasons are attributable to perceived stigma, poor mental health literacy, and negative attitudes toward seeking professionals [3,4].

As studies have demonstrated that different illnesses lead to distinctive patterns of web-based help seeking, it is crucial to examine the web-based behavior of individuals seeking help for specific illnesses such as depression [5,6]. In particular, chronic patients increasingly seek web-based help to self-manage their conditions [7-10], and, as in the case of depressive symptomatology, they prefer to discuss their illness with others with the same disease [11].

The ability to connect with others anonymously is an essential feature of online support groups, which enables them to overcome their fear of stigmatization [12,13]. The latter is particularly important in the context of mental health, as it is a notable barrier to seeking formal aid [3,14]. In addition, when compared with other mental health problems, people with depressive symptoms have different beliefs about their illness, resulting in diverse help-seeking trajectories, including long delays between the onset of symptoms and seeking help [5,6,15]. Notably, information gathering, social support, and empowerment in patients’ coping with depressive symptoms are important aspects to consider [16]. These were key aspects before the internet revolution of peer-to-peer support groups, but presumably, their accessibility and importance have changed since then.

In this review and meta-analysis, we aimed to systematically search and summarize the scientific literature on web-based help seeking and depressive symptoms and determine how web-based help-seeking behaviors are carried out in people with depressive symptomatology by considering both the antecedents and consequences of such behavior. On the basis of these findings, we propose an integrated framework that may help overcome the limitations of previous reviews on this topic.

### Depressive Symptoms and Web-Based Help Seeking

The search for web-based health information and support has become increasingly frequent. In 2020, 7 out of 10 Americans [17] obtained health-related information from the internet, and almost two-thirds of health consumers in the United States sought information on social media [18]. Similar estimates were found in Europe [19], where over half of those aged 16 to 74 years sought web-based help. The high accessibility, immediacy, and interactiveness generated by digital platforms are some of the reasons for the high prevalence of such behavior [20]. Mental health disorders are a crucial public health issue, with prevalence rates of depressive and anxiety symptoms steadily increasing. People with depressive symptoms are likely to seek health information on the internet [21,22]. However, investigating how has been challenging owing to the large variability of web-based information and behaviors [23-25]. On the one hand, people with depressive symptoms search for web-based help to become better informed about how to manage their health conditions [4,5,26-30]; however, web-based help seeking is not limited to seeking practical advice (ie, health information seeking) but also includes the emotional support that other users may solicit and provide, such as in online support groups [31,32]. One can actively or passively participate in online support groups, thus affecting depression levels. For example, people who lurk (ie, observe rather than participate [33]) are less likely to accrue benefits (eg, feel empowered) than those who actively participate. However, lurkers constitute a substantial proportion of online support groups, with estimates ranging from 45.5% to 90% [34]. In addition, active web-based interactions with others may not only help alleviate [35,36] but also worsen symptoms of depression owing to exposure to others’ negative emotions and corumination [37]. However, there is conflicting evidence on the extent to which people with depressive symptoms use web-based help-seeking services such as online support groups, how they use them (lurking vs posting activities), and the outcomes of such behavior.

Because it is difficult to distinguish between these 2 highly intertwined behaviors—information exchange and emotional support [38,39]—we will use the term “web-based help-seeking” throughout the manuscript to refer to both health information and emotional support seeking.

### Outcomes of Web-Based Help Seeking

Few studies have explored the outcomes of web-based help seeking in the context of general health [40,41]. For example, in a recent systematic review, Smailhodzic et al [42] found that social media use fostered equal communication between patients and health care professionals and empowered patients through improved disease management and control. Patient empowerment can be defined as the “feelings of power, control, and self-esteem that lead the patient to value autonomy—and thus interest in and desire to participate in healthcare decisions” [43]. Schulz and Nakamoto [43] applied the following four constructs (originally from Spreitzer [44]) to the context of health: (1) “Meaningfulness” refers to the experience that what a person is doing is meaningful for their health; (2) “Self-efficacy” is the belief in one’s capabilities to produce the desired results by one’s actions; (3) “Self-determination” refers to a decision that is characterized by an autonomous initiation; and (4) “Impact” is the difference that the accomplishment of a task is perceived to make in the scheme of things. Few studies have examined empowerment within the context of web-based help seeking [37,45,46]. In particular, in online support groups, individuals can empower each other by exchanging information, encountering emotional support, finding recognition, and helping others [7,47]. Opportunities for empowering people who seek web-based help for their mental health symptoms remain an object of research. In particular, the extent to which people with depressive symptoms feel empowered by web-based help seeking remains an open question.

### Previous Reviews on Depressive Symptoms and Web-Based Help Seeking

Previous systematic reviews have focused on the antecedents of web-based help-seeking behaviors as well as the different characteristics of web-based health channels [5,48-51]. Two recent systematic reviews and meta-analyses [48,51] found that contextual information (eg, quality and trust) of web-based information predicted web-based help-seeking behaviors better than other types of information (eg, attitudes toward seeking, perceived knowledge, and health literacy). However, these reviews investigated web-based help seeking in the context of general mental health problems, and no up-to-date reviews exist on web-based help seeking and depressive symptoms [52]. In particular, systematic reviews on web-based help seeking and depressive symptoms are outdated because they refer to online support groups [53-56] without considering the rapid growth of social media–based communication and support [57]. Online support groups and social media platforms may differ in terms of anonymity, participation rates, and social network opportunities. Furthermore, randomized controlled trials of web-based interventions, such as cognitive behavioral therapy [3,52,58], failed to capture the natural phenomenon of web-based help seeking outside the purpose of an intervention. In addition, previous systematic reviews reported inconsistent findings related to the benefits of social media use and social networking interventions in young people with depressive symptoms [59]. Indeed, differences may emerge among different generational cohorts because of the development of the digital landscape, with younger generations being more technologically savvy [60]. In this regard, younger generations have integrated technology into almost all areas of their lives, including web-based help-seeking behavior for mental health problems [61].

### Study Aim

In this review and meta-analysis, we aimed to systematically search and summarize the scientific literature on web-based help seeking and depressive symptoms and determine how web-based help-seeking behaviors are carried out in people with depressive symptomatology by considering both the antecedents and consequences of such behavior. On the basis of these findings, we propose an integrated framework that may help overcome the limitations of previous reviews on this topic.

## Methods

This systematic review was conducted according to the PRISMA (Preferred Reporting Items for Systematic Reviews and Meta-Analyses) statement [62]. The protocol was registered in PROSPERO (CRD42021250177).

### Search Strategy, Study Selection, and Data Extraction

A systematic literature search was carried out from their inception in 6 electronic databases (September 14, 2022): PsycINFO, MEDLINE, Embase (via Ovid), CINAHL, Communication & Mass Media Complete (via Ebscohost), and Web of Science (Clarivate Analytics) using key terms such as “depress*” OR “mood disord*” AND “help-seeking behavior” OR “information seeking” AND different types of study design like “surveys OR cross-sectional OR cohort*.” We did not restrict the search to a specific age range of participants. The detailed search strategy and keywords used are presented in Multimedia Appendix 1. Finally, in October 2022, a manual search was conducted of the reference lists of the included studies in Google Scholar (limited to the first 100 entries for different combinations of keywords used in the other databases).

We imported all entries in Zotero to automatically remove any duplicates. The first author and another coder (a Master student in Psychology and Health Communication) independently completed the title and abstract screening using Rayyan software [63]. To obtain a measure of interrater reliability, we calculated Cohen κ statistic to screen titles and abstracts (Cohen κ=0.84). Disagreements (n=34) were resolved through a consensus meeting with a third researcher (LM). To be included in this phase, a study had to meet the following eligibility criteria: (1) include a measure of depressive symptoms, (2) include a measure of web-based help-seeking behavior (such as website consultation or online support groups), and (3) report a measure of the association between depressive symptoms and web-based help-seeking behaviors. In addition, in the case of compound scales for mental health symptoms, articles were included solely if they reported a score on the depression subscale.

Studies reporting information on internet depression therapy, telephone depression counseling, or internet use outside health-related depression purposes were excluded. Studies were also excluded when their research methods were consistent in experiments, systematic reviews, or content analyses, or if they included a population with neurological disorders or inpatients. The latter limits clinical heterogeneity in participants. The first author then continued the full-text screening.

The following information was extracted from the included studies (list of included studies in Multimedia Appendix 2 [21,64-108]): study design and methodology, the country where the study was conducted, sample size, sex (% male), the average age of participants, type of recruitment (web-based vs other), depressive symptoms measure, the type of web-based help-seeking behavior (“online support group” vs “internet in general” vs “specific applications”), and key findings. Furthermore, to analyze changes over time, studies were classified according to generational cohorts [109] comprising Boomers I (born 1946-1954), Boomers II (1955-1964), Gen X (1965-1980), Gen Y or Millennials (1981-1996), and Gen Z (1997-2012). The detailed characteristics of all included studies are available in Multimedia Appendix 3. A summary of the extracted variables is presented in Multimedia Appendix 4. Data were reported in an MS Excel spreadsheet compiled by the first author and checked by LM. Missing data were handled by contacting the researchers for unreported data or additional details. In longitudinal studies, only the baseline information was extracted.

### Methodological Quality Assessment

To assess the methodological quality of each study, we used an adapted version of the Strengthening the Reporting of Observational studies in Epidemiology (STROBE) checklist [110]. In addition to the STROBE criteria, the following criteria were evaluated: (1) depressive symptom operationalization (0=self-reported diagnosis, 1=cutoff score on a depression rating scale, 2=diagnostic interview); (2) the presence of a reliability check for multiple-item measures of depression (1=present, 0=absent, NA=not applicable owing to single-item measures or clinical interviews); and (3) sampling strategy (1=random, 0=convenience). More information on the quality assessment of the studies can be found in Multimedia Appendix 5.

### Meta-analysis

Meta-analytic syntheses were carried out only for studies that included raw data convertible into effect sizes and similar concepts. The “meta” [111] package in R (R Foundation for Statistical Computing) statistical software was used for the meta-analysis. The Fisher r-to-z transformation was calculated as a measure of effect size, and the results were converted back to r correlation coefficients for interpretation. Conversion formulas and “esc” package [112,113] were used when necessary to convert raw data to correlations. Meta-analyses were performed to link depressive symptoms with online support group use, internet use in general, and self-help tools as primary outcomes. We also included the following two outcomes: (1) empowerment of the person and (2) type of formal service used after using the abovementioned web-based services as secondary outcomes. We interpreted pooled effect sizes of *r*<0.10, 0.10<*r*<0.50, and *r*>0.50 as small, medium, and large, respectively [114]. An inverse-variance method with a random-effects model and Hartung-Knapp-Sidik-Jonkman adjustment [114] were used to adjust for study differences in sample sizes. The heterogeneity of results was calculated using the between-study variance *τ*^2^, with the restricted maximum-likelihood estimator, and reported as the *I*^2^ statistic [112,115,116]. We interpreted the *I*^2^ level as low (25%), moderate (50%), and high (75%) [115]. When possible, additional meta-regression and subgroup analyses were carried out to investigate the role of moderators, such as generational cohort and sex of participants. Funnel plots and the Egger linear regression tests for funnel plot asymmetry were computed (when *k*≥10) to assess the presence of publication bias. Because of the paucity of studies investigating Generation Z, the article by Toscos [64] was grouped with the millennials category.

## Results

### Main Study Characteristics

**Figure 1:**
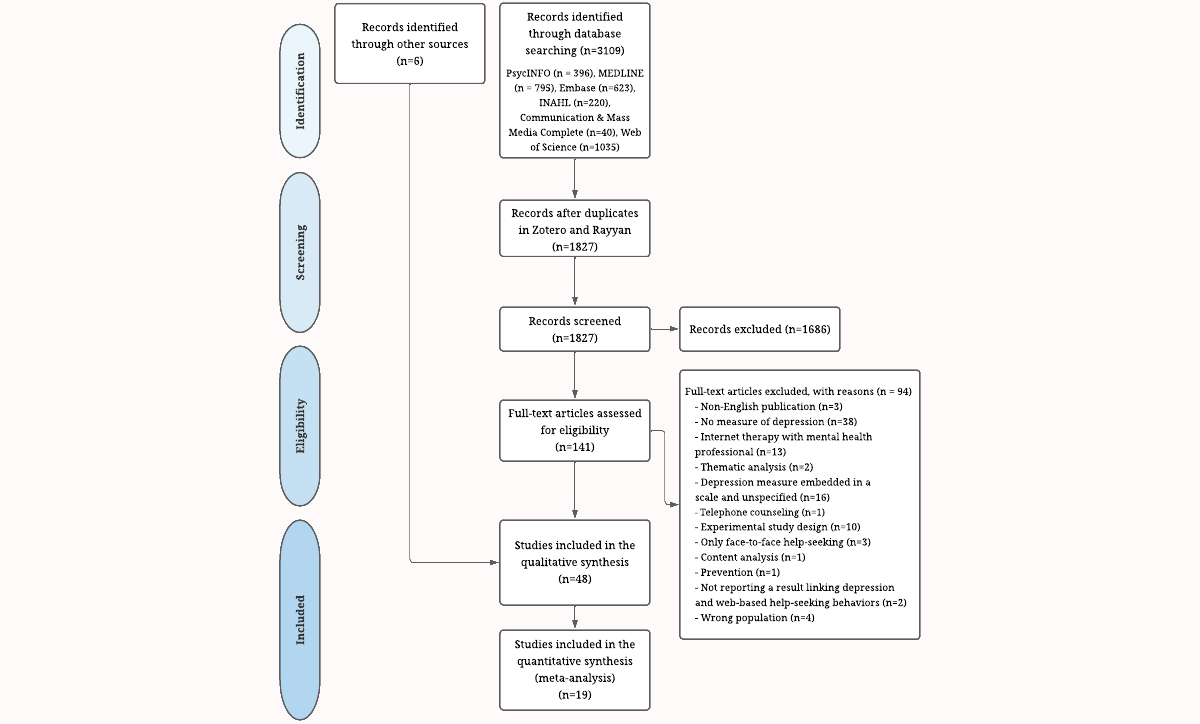
PRISMA (Preferred Reporting Items for Systematic Reviews and Meta-Analyses) flowchart.

As shown in the PRISMA flowchart (Figure 1), the database search resulted in 3109 publications, including 1282 duplicates. Following title and abstract screening, 141 full texts were screened for eligibility, resulting in 48 included studies. In addition, at the beginning of September 2022, a manual search was carried out in Google Scholar, looking at the first 100 entries, resulting in 6 other added articles. A total of 48 studies were included in the qualitative synthesis and 19 were included in the meta-analysis. The retained studies were published in 37 different journals between 2002 and 2022.

Before presenting the results of the studies, we acknowledge that they may be highly fragmentary owing to their heterogeneity. Hence, we have organized the description of the results as follows: the first part focuses on the descriptive characteristics of the included studies and the measures used for depressive symptoms. Then, the results will present the characteristics of web-based help seeking in terms of antecedents, patterns, and outcomes of behavior. Meta-analytic results will be reported following the related qualitative section.

### Methodological Quality Assessment

The articles’ quality scores ranged from 10 to 14 points (out of 15) based on the available study information (according to the STROBE checklist [110]). In particular, only 19% (9/48) of studies reported having applied a random sampling strategy. In addition, the number of items for multi-item indicators of depressive symptoms ranged from 2 to 20, and scale reliability, assessed with α, was reported in 17 studies (range .72<α<.94). Many (38/48, 79%) studies adopted a cross-sectional design, whereas only 21% (10/48) of studies used a longitudinal design. Most of the studies were conducted in Western countries, particularly Europe (14/48, 29%) and North America (16/48, 33%).

The analytical sample size varied greatly, with a median of 434.5, ranging from 32 [65, 66] to 264,431 participants [66]. On average, the mean percentage of male participants was 36.6%, which was calculated by excluding studies that looked specifically at the web-based help-seeking behavior of a specific population (eg, postpartum depression, adolescent girls, and patients with prostate cancer). The participants’ mean age was 37.03 years, ranging from 11 to 65 years. Studies predominantly investigated younger generations (Gen X, Y, Z; 33/48, 69%) rather than older generations (Boomers I and II; 14/48, 29%). Overall, 62% (30/48) of studies involved community-based adult samples, 21% (10/48) addressed caregivers or parents, and 12% (6/48) addressed patients with cancer.

### Overview of Web-Based Help Seeking and Depressive Symptoms

In line with previous research in the field [51], we distinguished different antecedents of web-based help seeking, such as psychological and instrumental factors. Psychological factors included intentions, attitudes, and beliefs. Intentions were assessed in 5 studies [67-71,117], stating that up to half of the respondents were generally willing to join online support groups [69,71,68] and that participants with higher depression scores were substantially more likely to express willingness to use web-based chat sites and self-help resources [64,67]. Concerning attitudes and beliefs toward web-based help seeking, 8 studies specifically addressed preferences in terms of comparison with offline help seeking—formal or informal—and showed that participants preferred face-to-face interventions over digital ones [69,72,73], although 1 study [73] found that internet-based information resources were the preferred support of Australian fathers. Furthermore, Leech et al [74] reported that male participants were more likely to seek web-based help. Gold et al [75] investigated differences in preferences among women participating in face-to-face versus online support groups. In total, 80% (47/60) of women in the face-to-face group, compared with only 31.2% (130/416) in the online support groups, agreed or strongly agreed that they would prefer to talk with someone in person than over the internet. Two studies [76,119] reported that web-based help seeking was a common way to avoid the stigma associated with mental illness. In addition, attitudes toward self-reliance, defined as the tendency to make decisions by themselves, have been reported as barriers to web-based help seeking [73,74,77].

Instrumental factors relate to the utility, trustworthiness, and credibility of web-based help-seeking resources and information. These were assessed in 15 studies, including measures of perceived usefulness or satisfaction with the message board. In 8 studies, participants trusted web-based information [21], which was judged as beneficial to manage their illness and allowed them to discuss the topic of depression more freely [28,67,72,79-83]. In addition, in a WeChat professionally facilitated support group, community management was considered good or very good [84]. Only in 1 study about multiple sclerosis, the reliability of website information was considered problematic according to the participants [85].

Interestingly, participants found relief not only in learning that they were not alone or “going crazy” [75] but also in knowing that their struggle could help others [86,91]. In only 1 study [118] that focused on mental health apps, participants expressed concerns about the accuracy and trustworthiness of the information reported in the app.

### Web-Based Help-Seeking Behaviors

Our findings showed that people use different types of technology to support their mental health. Hence, web-based help-seeking behaviors were divided into (1) online support groups, (2) internet use in general, and (3) self-help tools.

#### Online Support Groups

Most (41/48, 85%) studies focused on using online support groups such as forums or bulletin boards—previously existing or created ad hoc for research purposes—through which people with depressive symptoms can discuss their issues with others and receive advice. Importantly, starting from 2013, with the work of Wright [120], 23% (11/48) of studies investigated web-based communities present on social networking sites.

Approximately half (19/41, 46%) of the studies addressed the use of online support groups in terms of “length and frequency of visits” and one-fourth (7/41, 17%) in terms of “duration of membership.” Two included studies reported that patients with depression were among the most frequent users of the internet for health information with respect to other chronic illnesses [21,80]. Furthermore, 3 studies showed that those with a greater need for help (ie, higher depression scores) reported a greater use of web-based resources and engagement [78,67,120].

**Figure 2:**
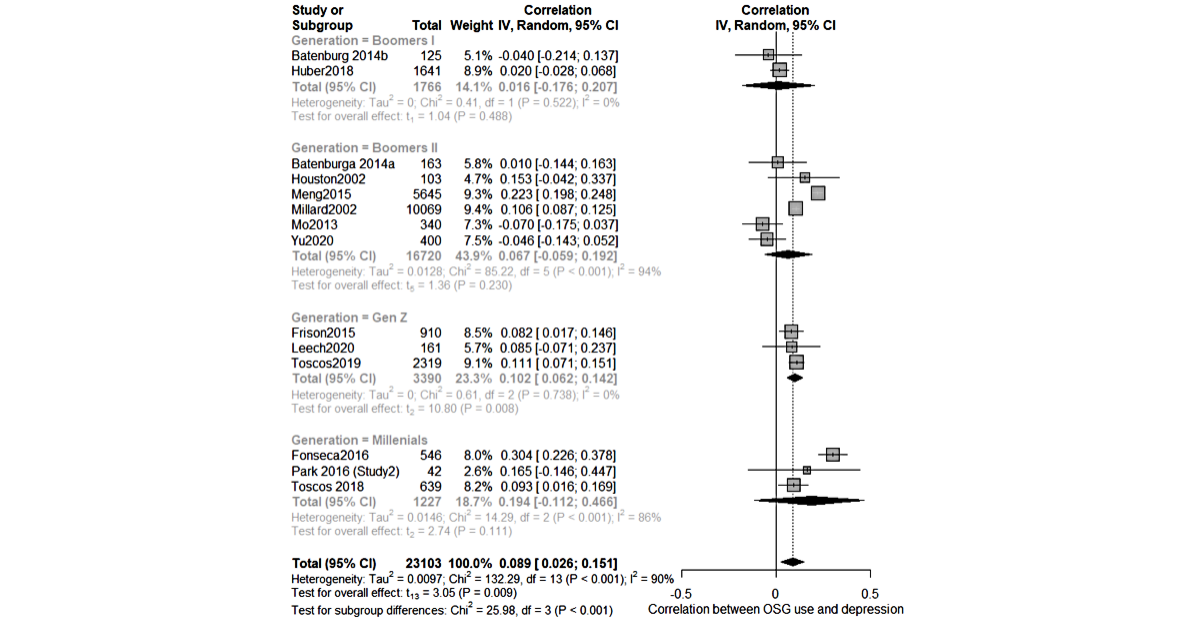
Forest plot of the meta-analysis on online support group use and depressive symptoms.

The meta-analysis results showed that overall, the link between depressive symptoms and web-based help-seeking behaviors through online support groups was positive but very small, with considerable heterogeneity (*k*=14; *r*=0.089, 95% CI 0.026-0.151; *P*=.009; *I*^2^=90%; Figure 2). Overall, less than 1% of help-seeking variation can be attributed to depression score variation. However, subgroup analyses showed significant differences in the effect size with respect to participants’ generations (*P*<.001). Indeed, the effect size was not significant when studies included participants from Boomers I (*k*=2; *r*=0.016, 95% CI −0.176 to 0.207; *P*=.49; *I*^2^=0%) and II (*k*=6; *r*=0.067, 95% CI −0.059 to 0.192; *P*=.23; *I*^2^=94%). The effect was larger but still not significant for Generation Y or millennials (*k*=3; *r*=0.194, 95% CI −0.112 to 0.466; *P*=.11; *I*^2^=86%) probably because of the high heterogeneity. The youngest participants, that is, Generation Z (*k*=3; *r*=0.102, 95% CI 0.062-0.142; *P*=.008; *I*^2^=0%) reported a small, positive, and significant effect size, meaning that they more frequently searched for mental health information with respect to people of other generations. The funnel plot (Multimedia Appendix 6) was symmetrical, and the results of the Egger test for funnel plot asymmetry were not significant (2-tailed *t* test, t_12_=−1.04; *P*=.32), thus indicating the absence of publication bias.

#### General Internet Use

A total of 17% (8/48) of studies assessed the general use of the internet in terms of website consultations [21,65,73,77,85,87,88,118,129]. To address web-based help-seeking behavior, most (5/8, 62%) studies used a single-item measure, such as “Have you ever used internet for seeking health information?” [21,77,85,87,88]. One study used a 5-point scale to investigate preferences for mental health support [73], and another looked at the type of information searched (eg, symptoms, treatment, or suicide) [65]. Findings in 1 study also mirror results from online support groups, that is, people with depressive symptoms were more likely to use the internet to find health information than people with other health conditions [21]. However, the meta-analysis linking internet use in general and depressive symptoms included very few studies, and the effect size was not significant (*k*=4; *r*=0.313, 95% CI −0.233 to 0.709; *P*=.16; *I*^2^=99%).

#### Self-help Tools

The last category included studies on self-help tools in terms of apps or websites, and 4 studies investigated this aspect [64,69,70,118]. Three studies showed that participants with higher levels of depression also reported greater use of these apps and considered them to be more appealing [70,87,118]. The meta-analysis included only 2 studies and a nonsignificant effect size (*k*=2; *r*=0.041, 95% CI −0.208 to 0.286; *P*=.28; *I*^2^=0%). Toscos et al [70] found that students who participated in their survey were more interested in self-help apps than in other types of web-based help. The authors suggest that this is because of the features of these tools, which are capable of overcoming barriers such as stigma and hesitance to self-disclose.

### Outcomes

The reported outcomes of web-based help seeking can be divided into the following: (1) changes in depressive symptomatology, (2) empowerment, and (3) formal service use*.* With respect to the former, 81% (34/48) of studies used self-report measures to assess depressive symptoms, whereas a few studies used diagnostic interviews (Multimedia Appendix 7). The most common measure to assess depression was the Center for Epidemiologic Studies Depression Scale (17/48, 35%) in various versions, not only in the original version from 1997 [89] (6/17, 35%) but also in the Iowa short form [121] (6/17, 35%).

**Figure 3:**
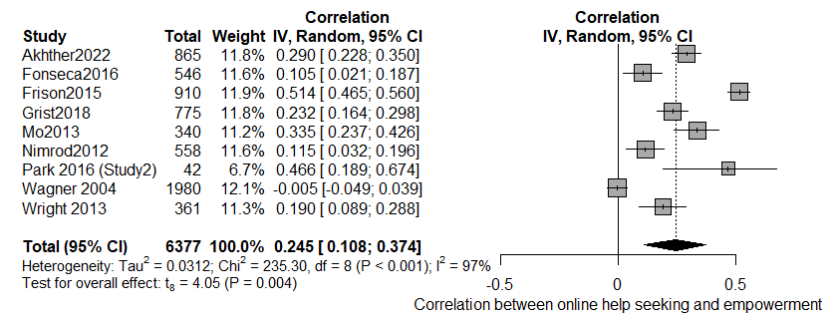
Forest plot of the meta-analysis on web-based help seeking and empowerment.

Furthermore, results regarding changes in depressive symptomatology were inconsistent. In total, 4 studies found that engagement in web-based activities was not associated with a decrease in depressive symptoms [90-93]; whereas, others found that participants with higher levels of online support group participation had a greater resolution of their symptomatology [71,72,81,93,123,124], sometimes indirectly through received and perceived social support or the experience of lower social isolation [92,124]. Only 1 study found that longer use time and session duration were associated with poorer depression outcomes [95]. The diversity of results was supported by a longitudinal study [80], which found that the positive effects of participating in online support groups were small for depressive symptoms. Similarly, in a study on WeChat [71], the daily time spent showed no substantial relationship with health outcomes. Patient empowerment was classified according to Schulz and Nakamoto [43] (Multimedia Appendix 8). The most prevalent constructs addressed in the included studies were *meaningfulness* (n=12) and *self-efficacy* (n=9). The meaningfulness of web-based health information was operationalized as an additional “know how” and was related to a better understanding of the illness and treatment options [21,81-84,92,97,120], primarily resulting from the web-based social support offered by digital communities [93,98,99]. In addition, self-efficacy was investigated in terms of a perceived enhanced ability to manage the illness and related symptoms [21,80,81,83-85,92,97,120] or a better ability to discuss topics [83]. Overall, web-based help seeking boosted the participants’ self-efficacy. However, Huber et al [96] found that face-to-face support group members (vs online support group members) felt better prepared for a physician’s appointment. These results were confirmed by meta-analytic results that showed that in general, people with depressive symptoms felt empowered after engaging in web-based help-seeking behaviors. The meta-analysis, including 8 studies assessing the effects of web-based help seeking, showed a medium effect size (*r*=0.245, 95% CI 0.107-0.374; *P*=.004; *I*^2^=96%; Figure 3). In addition, web-based help-seeking behaviors also influenced the relationship between patients and health care professionals and the patient’s propensity to seek formal services (psychotherapy or psychiatric treatment). Studies focused on patients and health care professionals [21,80-83,91,96] showed that using online support groups influences ongoing treatment [21,72,96] or exerted at least a minor impact on how participants communicated with their physician [72,80], up to a point of delaying seeking professional help for a small percentage (52/604, 8.6%) of the participants [83]. In contrast, 1 article [123] reported that the use frequency of the web-based community was correlated only with help-seeking intentions from formal sources. According to the authors, as almost 88% (308/350) of the participants had been medicated for their mental health problems, users were making use of it to look for something other than (or in addition to) pharmacological interventions to help them deal with their mental health issues. However, a meta-analysis linking web-based help seeking and formal service use was not carried out because of the paucity of studies examining the 2 constructs.

## Discussion

### Principal Findings

**Figure 4:**
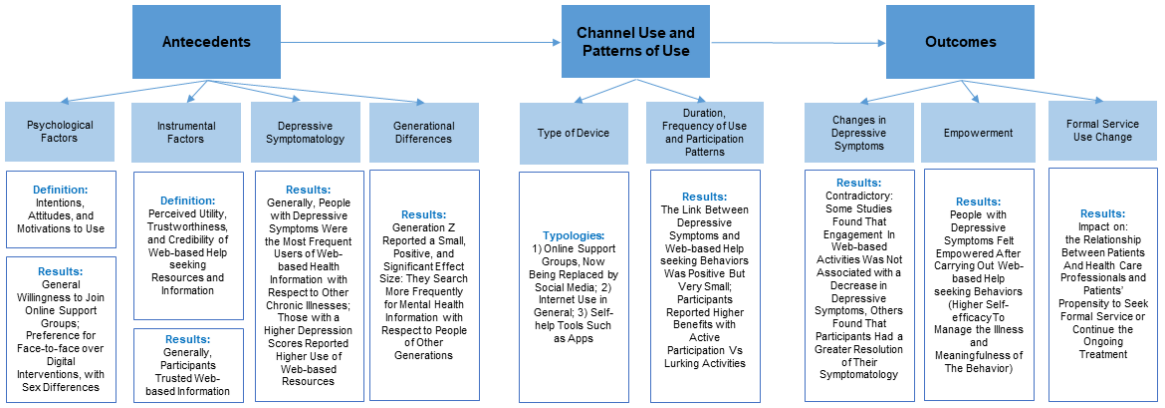
Proposed theoretical model.

**Figure 5:**
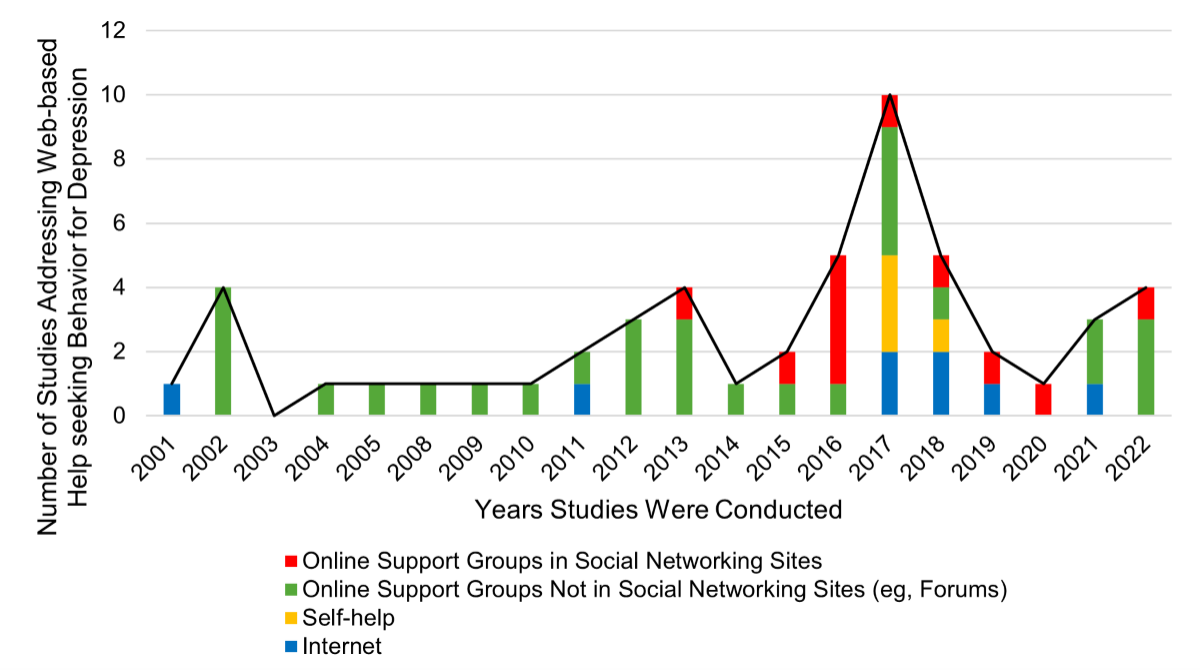
Overview of the timeline of the included studies.

Studies of web-based help-seeking behaviors in the context of depressive symptoms have increased extensively in recent decades. However, an up-to-date review and meta-analysis of the link between these 2 concepts is still lacking. On the basis of 48 studies, this study reveals important findings, which can be summarized hereunder and will be discussed in detail in the following paragraph: (1) web-based communities on social networking sites are replacing forums during the last few years, and people with depressive symptoms tend to rely on web-based help more often than before; (2) users with depressive symptoms (and with more severe symptomatology) reported higher use of web-based resources; and (3) the current literature investigated this phenomenon in a highly heterogeneous manner, including antecedents (eg, psychological and instrumental), patterns of use (eg, online support groups, general internet use, and self-help tools), and outcomes (eg, empowerment and seeking medical help). In addition, although the results showed that people with depressive symptomatology feel more empowered when seeking web-based help, (4) more outcomes of such behavior (especially iatrogenic consequences) should be considered and investigated. Finally, to guide future research, we propose a summary framework that reports all the concepts included in an organized structure (Figure 4). The first result mirrors the evolution of networking sites (Figure 5). In particular, until the advent of the first social network sites, such as Facebook in 2004 and Twitter in 2006 [99], online support groups found room on dedicated websites such as forums. Social networking sites have embedded online support groups in their features in various contexts related to health behavior or diseases, thus fostering participation rates and allowing access to online support groups at any time and location [125,126]. Note that social networking sites were not included in previous systematic reviews on the topic [55,56]. The meta-analysis results showed small positive effect sizes between depressive symptoms and web-based help-seeking behaviors, but the association was substantial only in the youngest generation, that is, Generation Z. Moreover, the qualitative summary provided evidence for the higher prevalence of people with depressive symptoms looking for information on the internet with respect to other chronic patients. This further stresses the importance of future studies on the investigation of web-based help seeking in young generations, which we proved are prone to managing their lives and health primarily on the internet. This is particularly relevant because new generations are heavy internet users [127]. Furthermore, we found great heterogeneity in the studies’ results and methodologies, which we tried to summarize into conceptually comparable categories, including antecedents and patterns of use of online support groups, the internet, or specific self-help tools such as apps. Despite the most recent systematic review and meta-analysis, according to which contextual information plays a more crucial role than psychological information [48,51], studies have investigated both psychological and contextual factors as antecedents. Despite the participants’ preference for asking for help from formal services such as mental health professionals, they made more use of informal web-based services with respect to the other options. This is probably because of the possibility of overcoming geographic location and time constraints to connect (anonymously) with others with similar experiences [128]. In addition, participation patterns play a role in health-related outcomes [129-131], where higher benefits were associated with posting (vs lurking) activities. Participants used online support groups to deal with depressive symptoms, as reported by the meta-analysis results. In addition, although the use of self-help tools such as mental health apps is a growing phenomenon [132], few studies have examined this form of web-based help seeking. In particular, measures of web-based help-seeking behavior should be refined. Studies have assessed this concept very differently, with only some making use of validated scales (eg, the Intensity of Online Support Group Participation scale by Batenburg et al [93]). Hence, future studies should rely on, and also develop, new measures of web-based help seeking by considering that this construct may cover both informational and socioemotional help simultaneously. As other authors noted earlier [41], more consistent methods to measure web-based help-seeking behaviors are required. The limitations of self-reported measures should be overcome by using mixed method approaches to investigate the types of posts and activities carried out in online support groups to better disentangle clusters in the use of social networking sites and, at the same time, acknowledge the multidimensional nature of web-based help seeking [120]. With respect to the outcomes, participants generally expressed that entering online support groups was beneficial and enhanced their mental health by promoting feelings of empowerment. Indeed, meta-analytic results showed that the link between empowerment and web-based help-seeking behaviors was positive and of medium size. This positive link is in line with previous systematic reviews that highlight the positive effects of web-based interventions on empowerment [133]. In addition, a recent study by Johansson et al [134] reported that participating in online support groups empowered patients to become more engaged and better contributed to the patient-provider relationship. However, the conceptualization of empowerment in these studies was vague; only 1 study explicitly mentioned empowerment [81]. Hence, as suggested by Barr et al [135], further work should develop a more appropriate measure to capture this construct, as it affects a broad range of health-related outcomes such as health status and medication adherence [136,137]. Measuring patient empowerment in people with depressive symptoms is crucial because it may influence self-stigma, one of the main barriers to seeking formal help [22,138]. In addition, more studies are needed to examine formal service use, a concept that has not been studied extensively. Owing to the scarcity of attention paid to this concept, it was not possible to make an informative summary in our study. However, empowerment is highly interconnected with formal service use and outcomes; empowerment not only increases self-management of chronic conditions but also influences the physician-patient relationship [139]. In some cases, the results were contradictory. In 1 experiment, Audrain-Pontevia and Menvielle [140] found that user empowerment was substantially but negatively related to user commitment to the physician. In contrast, McAllister et al [141], found that empowerment promoted a more collaborative approach to health care. In particular, it needs to be clarified whether web-based resources act as gateways offering opportunities for improving access to therapy for people with mental health problems or whether online support groups are experienced as substitutes of formal services. In addition, other factors should be considered when studying empowerment, such as health literacy. According to the Health Empowerment Model, empowering processes may not always lead to better health-related outcomes in patients with low health literacy [46,142]. Thus, we hypothesized that the positive or negative effects of web-based help-seeking behaviors may also depend on the level of health literacy. However, none of these studies assessed this variable. Ultimately, the proposed summary framework mirrors the categorization of variables reported in the included studies. As reported by previous theoretical models [51], antecedents can be differentiated into psychological or instrumental factors. In particular, considering that there may be differences in terms of accessibility and participation between online support groups from the past (eg, forums) and new communities (eg, social networking sites), our model considers and differentiates a variety of channels and pays close attention to participation patterns by distinguishing, for example, active participation from lurking activities. In addition, the model emphasizes outcomes by differentiating between empowering processes and service use changes. Further research on this topic should consider the stage of participants’ trajectory with respect to their illness and whether web-based help seeking affects their attitudes toward mental health professionals and treatment options. Future research should evaluate other possible unintended consequences, such as the fostering of cyberchondria, defined as an excessive pattern of web-based health research associated with an increase in health anxiety [143], a phenomenon frequently described in the context of the COVID-19 pandemic [144].

Gender differences should be considered when considering our model. For example, men may generally have higher levels of self-stigma, which, in turn, would affect their willingness to seek formal help [64,145]. In previous reviews [73,74], male participants were found to be more likely to seek informal support on the internet. This is arguably an important question that should be addressed in future research. In addition, further research is needed to explore the related implications of moving online support groups to social networking sites, especially considering the psychological and cognitive processes involved, as well as the antecedents and consequences. In addition, comorbidities should be controlled for specific psychopathological problems. Indeed, people with depressive symptoms often experience other psychopathological symptoms such as anxiety [146].

### Limitations

The limitations identified in this systematic review are mainly related to the considerable heterogeneity in participants’ conditions and assessments of web-based help seeking, including its antecedents and consequences. In addition, most studies were conducted in Europe and North America, leaving out the results from Asian, African, and South American countries. The meta-analytic synthesis of the results was limited because of the heterogeneity of the studies. In addition, only a few studies, such as websites or self-help tools, have investigated channels other than online support groups. Therefore, meta-analyses and subgroup comparisons were not possible for the different types of web-based help-seeking communication. Eventually, most of the included studies lacked a theoretical background to guide the discussion of concepts of interest.

### Conclusions

In conclusion, as web-based help-seeking behavior in the context of depressive symptoms is an ever-growing phenomenon, especially in younger generations, high-quality research is needed to investigate the effects of such behaviors in terms of benefits and pitfalls. A more rigorous and coherent assessment of the construct of interest that considers antecedents, patterns of use, and consequences is also needed. In addition, it would be useful to consider how different social networking sites, platforms, and their features and affordances could influence web-based seeking behaviors.

## Appendix

Multimedia Appendix 1 Search terms and strategy using Boolean operators.

Multimedia Appendix 2 Included studies.

Multimedia Appendix 3 Descriptive information of included articles.

Multimedia Appendix 4 Summary of variables in included articles.

Multimedia Appendix 5 Quality assessment of included articles.

Multimedia Appendix 6 Funnel plot.

Multimedia Appendix 7 Depression assessment.

Multimedia Appendix 8 Empowerment operationalization.

## Abbreviations

PRISMA: Preferred Reporting Items for Systematic Reviews and Meta-Analyses
STROBE: Strengthening the Reporting of Observational studies in Epidemiology
