# Supplementary material for: The Empowering Role of Web-Based Help Seeking on Depressive Symptoms: Systematic Review and Meta-analysis
Source: J Med Internet Res. 2023 Feb 2;25:e36964. doi: 10.2196/36964 (PMC9936373; doi:10.2196/36964)
Supplement: Multimedia Appendix 1 [file jmir_v25i1e36964_app1.docx]

Multimedia Appendix 1: Search Terms and Strategy Using Boolean Operators

This is a Multimedia Appendix to a full manuscript published in the J Med Internet Res. For full copyright and citation information see <http://dx.doi.org/10.219/3694>

**PsycINFO** *Ovid*

**#1 Depression**

affective disorders/ OR

anaclitic depression/ OR

"depression (emotion)"/ OR

dysthymic disorder/ OR

major depression/ OR

mental health/ OR

recurrent depression/ OR

reactive depression/ OR

seasonal affective disorder/ OR

(affective disorder* OR

affective symptom* OR

depress* OR

dysthymi* OR

MDD OR

MDE OR

mood disorder*).ti,ab,id.

**397.277 hits (April 14, 2021)**

**429.473 hits (September 14, 2022)**

**#2 Online help seeking**

(health care seeking behavior/ OR

help seeking behavior/ OR

information seeking/ OR

peer counseling/ OR

support groups/) AND

(internet* OR online*).ti,ab,id. OR

(communit* ADJ3 internet).ti,ab,id. OR

(communit* ADJ3 online).ti,ab,id. OR

(forum ADJ3 internet.ti,ab,id. OR

(forum ADJ3 online).ti,ab,id. OR

((assistance ADJ3 seeking).ti,ab,id. OR

(help ADJ3 seeking).ti,ab,id. OR

(information ADJ3 seeking).ti,ab,id. OR

(peer ADJ3 counsel*).ti,ab,id. OR

(peer ADJ3 support).ti,ab,id. OR

(seeking ADJ3 behavior).ti,ab,id. OR

(support ADJ3 group*).ti,ab,id.) AND

(internet* OR online*).ti,ab,id.

**8.617 hits (April 14, 2021)**

**9.948 hits (September 14, 2022)**

**#3 Study type**

cohort analysis/ OR

longitudinal studies/ OR

prospective studies/ OR

retrospective studies/ OR

surveys/ OR

(longitudinal study OR prospective study OR retrospective study).md. OR

cohort.ti,ab,id. OR

content analysis.ti,ab,id. OR

cross sectional.ti,ab,id. OR

crosssectional.ti,ab,id. OR

epidemiological stud*.ti,ab,id. OR

experiment* stud*.ti,ab,id. OR

longitudin*.ti,ab,id. OR

prevalence stud*.ti,ab,id. OR

prospective stud*.ti,ab,id. OR

retrospective stud*.ti,ab,id. OR

survey*.ti,ab,id.

**659.142 hits (April 14, 2021)**

**725.540 hits (September 14, 2022)**

**#1 AND #2 AND #3: 380 limit to Peer reviewed = 300 hits (April 14, 2021)**

**#1 AND #2 AND #3: 502 limit to Peer reviewed + yr=2021-current = 96 hits (September 14, 2022)**

**Medline *Ovid***

**#1 Depression**

adjustment disorder/ OR

depression/ OR

depressive disorder/ OR

depressive disorder, major/ OR

dysthymic disorder/ OR

mental health/ OR

mood disorders/ OR

seasonal affective disorder/ OR

(affective disorder* OR

affective symptom* OR

depress* OR

dysthymi* OR

MDD OR

MDE OR

mood disorder*).ti,ab,kf.

**584.785 hits (April 14, 2021)**

**646.143 hits (September 14, 2022)**

**#2 Online help seeking**

(help-seeking behavior/ OR

information seeking behavior/ OR

“patience acceptance of health care”/ OR

self-help groups/) AND

(internet* OR online*).ti,ab,kf. OR

(communit* ADJ3 internet).ti,ab,kf. OR

(communit* ADJ3 online).ti,ab,kf. OR

(forum ADJ3 internet).ti,ab,kf. OR

(forum ADJ3 online).ti,ab,kf. OR

((assistance ADJ3 seeking).ti,ab,kf. OR

(help ADJ3 seeking).ti,ab,kf. OR

(information ADJ3 seeking).ti,ab,kf. OR

(peer ADJ3 counsel*).ti,ab,kf. OR

(peer ADJ3 support).ti,ab,kf. OR

(seeking ADJ3 behavior).ti,ab,kf. OR

(support ADJ3 group*).ti,ab,kf.) AND

(internet* OR online*).ti,ab,kf.

**9.138 hits (April 14, 2021)**

**9.950 hits (September 14, 2022)**

**#3 Study type**

cohort studies/ OR

cross-sectional studies/ OR

longitudinal studies/ OR

prospective studies/ OR

retrospective studies/ OR

"surveys and questionnaires"/ OR

cohort.ti,ab,kf. OR

content analysis.ti,ab,kf. OR

cross sectional.ti,ab,kf. OR

crosssectional.ti,ab,kf. OR

epidemiological stud*.ti,ab,kf. OR

experiment* stud*.ti,ab,kf. OR

longitudin*.ti,ab,kf. OR

prevalence stud*.ti,ab,kf. OR

prospective stud*.ti,ab,kf. OR

retrospective stud*.ti,ab,kf. OR

survey*.ti,ab,kf.

**3.533.982 hits (April 14, 2021)**

**4.015.180 hits (September 14, 2022)**

**#1 AND #2 AND #3: 578 hits (April 14, 2021)**

**#1 AND #2 AND #3: 681 limit to yr=2021-current = 217 hits (September 14, 2022)**

**Embase *Ovid***

**#1 Depression**

adjustment disorder/ OR

depression/ OR

depressive disorder/ OR

depressive disorder, major/ OR

dysthymic disorder/ OR

mental health/ OR

mood disorders/ OR

seasonal affective disorder/ OR

(affective disorder* OR

affective symptom* OR

depress* OR

dysthymi* OR

MDD OR

MDE OR

mood disorder*).ti,ab,kw.

**967.388 hits (April 14, 2021)**

**1.060.784 hits (September 14, 2022)**

**#2 Online help seeking**

(help-seeking behavior/ OR

information seeking behavior/ OR

"patience acceptance of health care"/ OR

self-help groups/) AND

(internet* OR online*).ti,ab,kw. OR

(communit* ADJ3 internet).ti,ab,kw. OR

(communit* ADJ3 online).ti,ab,kw. OR

(forum ADJ3 internet).ti,ab,kw. OR

(forum ADJ3 online).ti,ab,kw. OR

((assistance ADJ3 seeking).ti,ab,kw. OR

(help ADJ3 seeking).ti,ab,kw. OR

(information ADJ3 seeking).ti,ab,kw. OR

(peer ADJ3 counsel*).ti,ab,kw. OR

(peer ADJ3 support).ti,ab,kw. OR

(seeking ADJ3 behavior).ti,ab,kw. OR

(support ADJ3 group*).ti,ab,kw.) AND

(internet* OR online*).ti,ab,kw.

**12.539 hits (April 14, 2021)**

**13.623 hits (September 14, 2022)**

**#3 Study type**

cohort analysis/ OR

cross-sectional study/ OR

health survey/ OR

longitudinal study/ OR

prospective study/ OR

retrospective study/ OR

cohort.ti,ab,kw. OR

content analysis.ti,ab,kw. OR

cross sectional.ti,ab,kw. OR

crosssectional.ti,ab,kw. OR

epidemiological stud*.ti,ab,kw. OR

experiment* stud*.ti,ab,kw. OR

longitudin*.ti,ab,kw. OR

prevalence stud*.ti,ab,kw. OR

prospective stud*.ti,ab,kw. OR

retrospective stud*.ti,ab,kw. OR

survey*.ti,ab,kw.

**4.381.035 hits (April 14, 2021)**

**5.056.712 hits (September 14, 2022)**

**#1 AND #2 AND #3: 873 limit to Embase = 409 hits (April 14, 2021)**

**#1 AND #2 AND #3: 1.100 hits limit to records from Embase + yr=2021-current = 214 hits (September 14, 2022)**

**CINAHL *Ebsco***

**#1 Depression**

((MH "adjustment disorders") OR (MH "affective disorders") OR (MH "depression") OR (MH "depression, reactive") OR (MH "dysthymic disorder") OR (MH "mental health") OR (MH "seasonal affective disorder") OR AB ("affective disorder*" OR "affective symptom*" OR depress* OR dysthymi* OR MDD OR MDE OR "mood disorder*") OR TI ("affective disorder*" OR "affective symptom*" OR depress* OR dysthymi* OR MDD OR MDE OR "mood disorder*"))

**226.274 hits (April 14, 2021)**

**255.715 hits (September 14, 2022)**

**#2 Online help seeking**

((MH "help seeking behavior") OR (MH "information seeking behavior") OR (MH "peer counseling") OR (MH "support groups")) AND (AB (internet* OR online*) OR TI (internet* OR online*)) OR

AB (communit* N3 internet OR communit* N3 online OR forum N3 internet OR forum N3 online) OR TI (communit* N3 internet OR communit* N3 online OR forum N3 internet OR forum N3 online) OR

AB ((assistance N3 seeking OR help N3 seeking OR information N3 seeking OR peer N3 counsel* OR peer N3 support OR seeking N3 behavior OR support N3 group*) AND (internet* OR online*)) OR TI ((assistance N3 seeking OR help N3 seeking OR information N3 seeking OR peer N3 counsel* OR peer N3 support OR seeking N3 behavior OR support N3 group*) AND (internet* OR online*))

**6.926 hits (April 14, 2021)**

**8.226 hits (September 14, 2022)**

**#3 Study type**

(MH "cross sectional studies") OR (MH "panel studies") OR (MH "prospective studies") OR (MH "retrospective panel studies") OR (MH "surveys") OR AB (cohort OR "content analysis" OR "cross sectional" OR crosssectional OR "epidemiological stud*" OR "experiment* stud*" OR longitudin* OR "prevalence stud*" OR "prospective stud*" OR "retrospective stud*" OR survey*) OR TI (cohort OR "content analysis" OR "cross sectional" OR crosssectional OR "epidemiological stud*" OR "experiment* stud*" OR longitudin* OR "prevalence stud*" OR "prospective stud*" OR "retrospective stud*" OR survey*)

**1.145.484 hits (April 14, 2021)**

**1.292.767 hits (September 14, 2022)**

**#1 AND #2 AND #3: 308 limit to Peer reviewed + Exclude Medline records = 144 hits (April 14, 2021)**

**#1 AND #2 AND #3: 411 limit to Peer reviewed + Exclude Medline records + Published date 01012021- = 76 hits (September 14, 2021)**

**Communication & Mass Media Complete *Ebsco***

**#1 Depression**

AB ("adjustment disorder*" OR "affective disorder*" OR "affective symptom*" OR depress* OR dysthymi* OR MDD OR MDE OR "mood disorder*") OR KW ("adjustment disorder*" OR "affective disorder*" OR "affective symptom*" OR depress* OR dysthymi* OR MDD OR MDE OR "mood disorder*") OR SU ("adjustment disorder*" OR "affective disorder*" OR "affective symptom*" OR depress* OR dysthymi* OR MDD OR MDE OR "mood disorder*") OR TI ("adjustment disorder*" OR "affective disorder*" OR "affective symptom*" OR depress* OR dysthymi* OR MDD OR MDE OR "mood disorder*")

**1.749 hits (April 14, 2021)**

**2.208 hits (September 14, 2022)**

**#2 Online help seeking**

AB (communit* N3 internet OR communit* N3 online OR forum N3 internet OR forum N3 online) OR KW (communit* N3 internet OR communit* N3 online OR forum N3 internet OR forum N3 online) OR SU (communit* N3 internet OR communit* N3 online OR forum N3 internet OR forum N3 online) OR TI (communit* N3 internet OR communit* N3 online OR forum N3 internet OR forum N3 online) OR

AB ((assistance N3 seeking OR help N3 seeking OR information N3 seeking OR peer N3 counsel* OR peer N3 support OR seeking N3 behavior OR support N3 group*) AND (internet* OR online*)) OR KW ((assistance N3 seeking OR help N3 seeking OR information N3 seeking OR peer N3 counsel* OR peer N3 support OR seeking N3 behavior OR support N3 group*) AND (internet* OR online*)) OR SU ((assistance N3 seeking OR help N3 seeking OR information N3 seeking OR peer N3 counsel* OR peer N3 support OR seeking N3 behavior OR support N3 group*) AND (internet* OR online*)) OR TI ((assistance N3 seeking OR help N3 seeking OR information N3 seeking OR peer N3 counsel* OR peer N3 support OR seeking N3 behavior OR support N3 group*) AND (internet* OR online*))

**3.057 hits (April 14, 2021)**

**3.879 hits (September 14, 2022)**

**#3 Study type**

**N.A.**

**#1 AND #2: 50 limit to Peer reviewed = 37 hits (April 14, 2021)**

**#1 AND #2: 57 limit to Peer reviewed + Published date 01012021- = 3 hits (September 14, 2022)**

**Web of Science *Clarivate Analytics***

**#1 Depression**

TS=("adjustment disorder*" OR "affective disorder*" OR "affective symptom*" OR depress* OR dysthymi* OR MDD OR MDE OR "mood disorder*")

**668.913 hits (April 14, 2021)**

**768.098 hits (September 14, 2022)**

**#2 Online help seeking**

TS=(communit* NEAR/3 internet OR communit* NEAR/3 online OR forum NEAR/3 internet OR forum N3 online) OR

TS=((assistance NEAR/3 seeking OR help NEAR/3 seeking OR information NEAR/3 seeking OR peer NEAR/3 counsel* OR peer NEAR/3 support OR seeking NEAR/3 behavior OR support NEAR/3 group*) AND (internet* OR online*))

**18.261 hits (April 14, 2021)**

**24.504 hits (September 14, 2022)**

**#3 Study type**

TS=(cohort OR "content analysis" OR "cross sectional" OR crosssectional OR "epidemiological stud*" OR "experiment* stud*" OR longitudin* OR "prevalence stud*" OR "prospective stud*" OR "retrospective stud*" OR survey*)

**3.069.501 hits (April 14, 2021)**

**3.696.185 hits (September 14, 2022)**

**#1 AND #2 AND #3: 719 hits (April 14, 2021)**

**#1 AND #2 AND #3: 1.028 limit to Publication date 2021-01-01 to 2022-09-14 = 316 hits (September 14, 2022)**
