# Supplementary material for: The Empowering Role of Web-Based Help Seeking on Depressive Symptoms: Systematic Review and Meta-analysis
Source: J Med Internet Res. 2023 Feb 2;25:e36964. doi: 10.2196/36964 (PMC9936373; doi:10.2196/36964)
Supplement: Multimedia Appendix 2 [file jmir_v25i1e36964_app2.docx]

Multimedia Appendix 2: Included studies

This is a Multimedia Appendix to a full manuscript published in the J Med Internet Res. For full copyright and citation information see <http://dx.doi.org/10.219/3694>

The following list contains the references for all 48 reviewed studies (as they appear in the reference list of the manuscript) included with a shortened denomination in brackets corresponding to the denomination used in the tables in other Multimedia Appendices.

Toscos, T., Coupe, A., Flanagan, M., Drouin, M., Carpenter, M., Reining, L., ... & Mirro, M. J. (2019). Teens using screens for help: Impact of suicidal ideation, anxiety, and depression levels on youth preferences for telemental health resources. *JMIR mental health, 6*(6), e13230. <https://doi.org/10.2196/13230> [Toscos2019]

Van Meter, A. R., Birnbaum, M. L., Rizvi, A., & Kane, J. M. (2019). Online help-seeking prior to diagnosis: Can web-based resources reduce the duration of untreated mood disorders in young people?. *Journal of affective disorders*, 252, 130-134. <https://doi.org/10.1016/j.jad.2019.04.019> [VanMeter2019]

DeAndrea, D. C., & Anthony, J. C. (2013). Online peer support for mental health problems in the United States: 2004–2010. *Psychological Medicine, 43*(11), 2277-2288. [https://doi.org/10.1017/S0033291713000172](https://psycnet.apa.org/doi/10.1017/S0033291713000172) [DeAndrea2013]

Fonseca, A., Gorayeb, R., & Canavarro, M. C. (2016). Women’s use of online resources and acceptance of e-mental health tools during the perinatal period. *International journal of medical informatics, 94*, 228-236. <https://doi.org/10.1016/j.ijmedinf.2016.07.016> [Fonseca2016]

Köhle, N., Drossaert, C. H., Van Uden-Kraan, C. F., Schreurs, K. M., Hagedoorn, M., Verdonck-de Leeuw, I. M., & Bohlmeijer, E. T. (2018). Intent to use a web-based psychological intervention for partners of cancer patients: associated factors and preferences. *Journal of psychosocial oncology, 36*(2), 203-221. <https://doi.org/10.1080/07347332.2017.1397831>[Kohle2018]

March, S., Day, J., Ritchie, G., Rowe, A., Gough, J., Hall, T., ... & Ireland, M. (2018). Attitudes toward e-mental health services in a community sample of adults: online survey. *Journal of medical Internet research, 20*(2), e59. <https://doi.org/10.2196/jmir.9109> [March2018]

Toscos, T., Carpenter, M., Drouin, M., Roebuck, A., Kerrigan, C., & Mirro, M. (2018). College Students' Experiences with, and Willingness to Use, Different Types of Telemental Health Resources: Do Gender, Depression/Anxiety, or Stress Levels Matter?. *Telemedicine and e-Health, 24*(12), 998-1005. <https://doi.org/10.1089/tmj.2017.0243> [Toscos2018]

Yu, Y., Li, Y., Li, T., Xi, S., Xiao, X., Xiao, S., & Tebes, J. K. (2020). New Path to Recovery and Well-Being: Cross-Sectional Study on WeChat Use and Endorsement of WeChat-Based mHealth Among People Living With Schizophrenia in China. *Journal of Medical Internet Research, 22*(9), e18663. <https://doi.org/10.2196/18663> [Yu2020]

Houston, T. K., Cooper, L. A., & Ford, D. E. (2002). Internet support groups for depression: a 1-year prospective cohort study. *American Journal of Psychiatry, 159*(12), 2062-2068.  [https://doi.org/10.1176/appi.ajp.159.12.2062](https://psycnet.apa.org/doi/10.1176/appi.ajp.159.12.2062) [Houston2002]

Giallo, R., Dunning, M., & Gent, A. (2017). Attitudinal barriers to help-seeking and preferences for mental health support among Australian fathers. *Journal of reproductive and infant psychology, 35*(3), 236-247. <https://doi.org/10.1080/02646838.2017.1298084> [Giallo2017]

Leech, T., Dorstyn, D. S., & Li, W. (2019). eMental health service use among Australian youth: a cross-sectional survey framed by Andersen’s model. *Australian Health Review, 44*(6), 891-897. <https://doi.org/10.1071/AH19095> [Leech2020]

Gold, K. J., Normandin, M. M., & Boggs, M. E. (2016). Are participants in face-to-face and internet support groups the same? Comparison of demographics and depression levels among women bereaved by stillbirth. *Archives of women's mental health, 19*(6), 1073-1078. <https://doi.org/10.1007/s00737-016-0657-x> [Gold2016]

Simmons, L. A., Wu, Q., Yang, N., Bush, H. M., & Crofford, L. J. (2015). Sources of health information among rural women in Western Kentucky. *Public Health Nursing, 32*(1), 3-14. <https://doi.org/10.1111/phn.12134> [Simmons2015]

McNair, R. P., & Bush, R. (2016). Mental health help seeking patterns and associations among Australian same sex attracted women, trans and gender diverse people: a survey-based study. *BMC psychiatry, 16*(1), 1-16. <https://doi.org/10.1186/s12888-016-0916-4> [McNair2016]

Akhther, N., & Sopory, P. (2022). Seeking and Sharing Mental Health Information on Social Media During COVID-19: Role of Depression and Anxiety, Peer Support, and Health Benefits. *Journal of Technology in Behavioral Science*, 1-16. <https://doi.org/10.1007/s41347-021-00239-x> [Akhter2022]

Kramer, J., Boon, B., Schotanus-Dijkstra, M., van Ballegooijen, W., Kerkhof, A., & Van Der Poel, A. (2015). The mental health of visitors of web-based support forums for bereaved by suicide*. Crisis*. <https://doi.org/10.1027/0227-5910/a000281> [Kramer2015]

Millard, R. W., & Fintak, P. A. (2002). Use of the Internet by patients with chronic illness. *Disease Management and Health Outcomes, 10*(3), 187-194. [Millard2002]

Mo, P. K., & Coulson, N. S. (2013). Online support group use and psychological health for individuals living with HIV/AIDS. *Patient Education and Counseling, 93*(3), 426-432. <https://doi.org/10.1016/j.pec.2013.04.004> [Mo2013]

Nimrod, G. (2013). Online depression communities: members' interests and perceived benefits. *Health communication, 28*(5), 425-434. <https://doi.org/10.1080/10410236.2012.691068> [Nimrod2013]

Powell, J., McCarthy, N., & Eysenbach, G. (2003). Cross-sectional survey of users of Internet depression communities. *BMC psychiatry, 3*(1), 1-7. <https://doi.org/10.1186/1471-244X-3-19> [Powell2003]

Han J, Guo G, Hong L. Impact of professionally facilitated peer support for family carers of people with dementia in a WeChat virtual community. *Journal of Telemedicine and Telecare*. 2022;28(1):68-76. https://doi.org/[10.1177/1357633X20910830](https://doi.org/10.1177/1357633X20910830) [Han2022]

Higueras, Y., Salas, E., Meca-Lallana, V., Rueda, P. C., De la Fuente, O. R., Cabello-Moruno, R., ... & Díaz, M. Á. R. (2022). Information-Seeking Strategies of People with Multiple Sclerosis in Spain: The INFOSEEK-MS Study. *Patient preference and adherence*, *16*, 51. <https://doi.org/10.2147/PPA.S344690> [Higueras2022]

Oh, Y. S., & Song, N. K. (2017). Investigating relationships between health-related problems and online health information seeking. *CIN: Computers, Informatics, Nursing, 35*(1), 29-35. https://doi.org/[10.1097/cin.0000000000000234](https://doi.org/10.1097/cin.0000000000000234) [Oh2017]

McKechnie, V., Barker, C., & Stott, J. (2014). The effectiveness of an Internet support forum for carers of people with dementia: a pre-post cohort study. *Journal of Medical Internet Research, 16*(2), e68. https://doi.org/[10.2196/jmir.3166](https://doi.org/10.2196/jmir.3166) [McKechnie2014]

Nimrod, G. (2012a). The membership life cycle in online support groups. *International Journal of Communication, 6*, 23. [Nimrod2012a]

Trail, T., Friedman, E., Rutter, C. M., & Tanielian, T. (2020). The relationship between engagement in online support groups and social isolation among military caregivers: longitudinal questionnaire study. *Journal of medical Internet research, 22*(4), e16423. https://doi.org/[10.2196/16423](https://doi.org/10.2196/16423) [Trail2020]

Batenburg, A., & Das, E. (2014a). Emotional approach coping and the effects of online peer-led support group participation among patients with breast cancer: a longitudinal study. *Journal of medical Internet research, 16*(11), e256. <https://doi.org/10.2196/jmir.3517> [Batenburga2014]

Wagner, T. H., & Hibbard, J. H. (2001). Who uses self-care books, advice nurses, and computers for health information?. *International journal of technology assessment in health care*, *17*(4), 590-600. <https://doi.org/10.1089/tmj.2017.0243> PMID: 11758302 [Wagner2004]

Marinova, N., Rogers, T., & MacBeth, A. (2022). Predictors of Adolescent Engagement and Outcomes–a cross-sectional study using the Togetherall (formerly Big White Wall) digital mental health platform. *Journal of Affective Disorders*. <https://doi.org/10.1016/j.jad.2022.05.058> [Marinova2022]

Huber, J., Muck, T., Maatz, P., Keck, B., Enders, P., Maatouk, I., & Ihrig, A. (2018). Face-to-face vs. online peer support groups for prostate cancer: a cross-sectional comparison study. *Journal of Cancer Survivorship, 12*(1), 1-9. <https://doi.org/10.1007/s11764-017-0633-0> [Huber2018]

Brailovskaia, J., & Margraf, J. (2016). Comparing Facebook users and Facebook non-users: Relationship between personality traits and mental health variables–an exploratory study. *PloS one, 11*(12), e0166999. <https://doi.org/10.1371/journal.pone.0166999> [Brailovskaia2016]

Park, J., Lee, D. S., Shablack, H., Verduyn, P., Deldin, P., Ybarra, O., ... & Kross, E. (2016). When perceptions defy reality: the relationships between depression and actual and perceived Facebook social support. *Journal of Affective Disorders*, *200*, 37-44. <https://doi.org/10.1016/j.jad.2016.01.048> [Park2016]

Algtewi, E., Owens, J., & Baker, S. R. (2017). Online support groups for head and neck cancer and health-related quality of life. *Quality of life research : an international journal of quality of life aspects of treatment, care and rehabilitation, 26*(9), 2351–2362.  [https://doi.org/10.1007/s11136-017-1575-8](https://psycnet.apa.org/doi/10.1007/s11136-017-1575-8) [Algtewi2017]

Klemm, P., & Hardie, T. (2007). Depression in Internet and face-to-face cancer support groups: a pilot study. *Oncology nursing forum, 29*(4), E45-E51. <https://doi.org/10.1188/02.ONF.E45-E51> [Klemm2002]

Kumar, S., Tran, J. L., Ramirez, E., Lee, W. N., Foschini, L., & Juusola, J. L. (2020). Design, recruitment, and baseline characteristics of a virtual 1-year mental health study on behavioral data and health outcomes: observational Study. *JMIR Mental Health, 7*(7), e17075. https://doi.org/[10.2196/17075](https://doi.org/10.2196/17075) [Kumar2020]

Lieberman, M. A., & Goldstein, B. A. (2005). Self-help on-line: an outcome evaluation of breast cancer bulletin boards. *Journal of Health Psychology, 10*(6), 855-862. [https://doi.org/10.1177/1359105305057319](https://psycnet.apa.org/doi/10.1177/1359105305057319) [Lieberman2005]

Meng, X., D’Arcy, C., & Adams, G. C. (2015). Associations between adult attachment style and mental health care utilization: Findings from a large-scale national survey. *Psychiatry research, 229*(1-2), 454-461. <https://doi.org/10.1016/j.psychres.2015.05.092> [Meng2015]

Merchant, R., Goldin, A., Manjanatha, D., Harter, C., Chandler, J., Lipp, A., ... & Naslund, J. A. (2022). Opportunities to expand access to mental health services: A case for the role of online peer support communities. *Psychiatric Quarterly*, *93*(2), 613-625. <https://doi.org/10.1007/s11126-022-09974-7> [Merchant2022]

Naslund, J. A., Aschbrenner, K. A., Marsch, L. A., & Bartels, S. J. (2016). The future of mental health care: peer-to-peer support and social media. *Epidemiology and psychiatric sciences*, *25*(2), 113-122. <https://doi.org/10.1017/S2045796015001067> [Naslund2019]

Nimrod, G. (2012b). Online Depression Communities: Does Gender Matter?. *Cyberpsychology: Journal of Psychosocial Research on Cyberspace, 6*(1). <https://doi.org/10.5817/CP2012-1-6> [Nimrod2012b]

Roystonn, K., Vaingankar, J. A., Chua, B. Y., Sambasivam, R., Shafie, S., Jeyagurunathan, A., ... & Subramaniam, M. (2020). The Public Health Impact and Policy Implications of Online Support Group Use for Mental Health in Singapore: Cross-Sectional Survey. *JMIR mental health, 7*(8), e18114. <https://doi.org/10.2196/18114> [Roystonn2020]

Grist, R., Cliffe, B., Denne, M., Croker, A., & Stallard, P. (2018). An online survey of young adolescent girls' use of the internet and smartphone apps for mental health support. *BJPsych open, 4*(4), 302-306. <https://doi.org/10.1192/bjo.2018.43> [Grist2018]

Teaford, D., Goyal, D., & McNeish, S. G. (2015). Identification of postpartum depression in an online community. *Journal of Obstetric, Gynecologic & Neonatal Nursing, 44*(5), 578-586. <https://doi.org/10.1111/1552-6909.12740> [Teaford2015]

Wright, K. B., Rosenberg, J., Egbert, N., Ploeger, N. A., Bernard, D. R., & King, S. (2013). Communication competence, social support, and depression among college students: A model of Facebook and face-to-face support network influence*. Journal of health communication, 18*(1), 41-57. <https://doi.org/10.1080/10810730.2012.688250> [Wright2013]

Batenburg, A., & Das, E. (2014b). Emotional coping differences among breast cancer patients from an online support group: a cross-sectional study*. Journal of medical Internet research, 16*(2), e28. <https://doi.org/10.2196/jmir.2831> [Batenburgb2014]

Kobori, O., & Yoshinaga, N. (2021). Investigation of the Effects of an Online Support Group for Mental Health Problems on Stigma and Help-Seeking Among Japanese Adults: Cross-sectional Study. *JMIR Formative Research*, *5*(9), e21348. <https://doi.org/10.2196/21348> [Kobori2021]

Frison, E., & Eggermont, S. (2015). The impact of daily stress on adolescents’ depressed mood: The role of social support seeking through Facebook. *Computers in Human Behavior, 44*, 315-325. [https://doi.org/10.1016/j.chb.2014.11.070](https://psycnet.apa.org/doi/10.1016/j.chb.2014.11.070) [Frison2015]
