# Supplementary material for: The Empowering Role of Web-Based Help Seeking on Depressive Symptoms: Systematic Review and Meta-analysis
Source: J Med Internet Res. 2023 Feb 2;25:e36964. doi: 10.2196/36964 (PMC9936373; doi:10.2196/36964)
Supplement: Multimedia Appendix 3 [file jmir_v25i1e36964_app3.docx]

Multimedia Appendix 3: Descriptive information
This is a Multimedia Appendix to a full manuscript published in the J Med Internet Res. For full copyright and citation information see <http://dx.doi.org/10.219/3694>

| Study ID | Journal | Country | Design* | Sample size | Recruitment** | Type of sampling*** | Male (%) | Age |
| --- | --- | --- | --- | --- | --- | --- | --- | --- |
| Akhther2022 | *Journal of Technology in Behavioral Science* | USA | C | 865 | 2 | R | 51.00% | 33.5 (21.36) |
| Algtewi2017 | *Quality of Life Research* | Mixed | C | 199 | 1 | C | 49.20% | 54.34 (SD=10.2) |
| Batenburga2014a | *Journal of Medical Internet Research* | Netherlands | C | 184 | 1 | C | 0.00% | 48.09 (SD =9.04) |
| Batenburgb2014b | *Journal of Medical Internet Research* | Netherlands | L | 133 | 1 | C | 0.00% | 48.44 (SD = 8.60) |
| Brailovskaia2016 | *PloS one* | Germany | C | 945 | 1 | C | 36.00% | 23.72 |
| DeAndrea2013 | *Psychological Medicine* | USA | L | 264431 | 1 | R | 47.00% | n/a |
| Fonseca2016 | *International journal of medical informatics* | Portugal | C | 546 | 1 | C | 0.00% | 31.55 (SD = 4.09) |
| Frison2015 | *Computers in Human Behavior* | Belgium | C | 910 | 0 | C | 48.10% | 15.44 (SD = 1.71) |
| Giallo2017 | *Journal of Reproductive and Infant Psychology* | Australia | C | 154 | 1 | C | 100.00% | 37.01 (SD = 6.57) |
| Gold2016 | *Archives of Women's Mental Health* | USA | C | 476 | 2 | C | 0.00% | 31 (SD =5.5) |
| Grist2018 | *BJPsych Open* | United Kingdom | C | 775 | 1 | C | 0.00% | 11–16 |
| Han2020 | *Journal of telemedicine and telecare* | China | L | 159 | 2 | C | 18.20% | 55 |
| Higueras2021 | *Patient Preference and Adherence* | Spain | C | 302 | 0 | C | 36% | 42.3 (10) |
| Houston2002 | *The American Journal of Psychiatry* | Mixed | L | 103 | 1 | C | 21.40% | 40 (median) |
| Huber2018 | *Journal of Cancer Survivorship* | Germany | C | 1641 | 1 | C | 1 | 68.4 |
| Klemm2002 | *Oncology nursing forum* | Mixed | C | 40 | 2 | C | 35.00% | n/a |
| Kobori2021 | *JMIR Form Res* | Japan | C | 350 | 1 | C | 33.71% | 38.36 (10.09) |
| Kohle2018 | *Journal of Psychosocial Oncology* | Netherlands | C | 168 | 2 | C | 52.00% | 59.2 (SD = 11.5) |
| Kramer2015 | *Crisis* | Netherlands and Dutch-speaking Belgium | L | 270 | 1 | C | 12.80% | 42.9 (SD = 12.4) |
| Kumar2020 | *JMIR mental health* | USA | L | 1288 | 1 | C | 22.00% | 33 (SD = 9) |
| Leech2020 | *Australian health review : a publication of the Australian Hospital Association* | Australia | C | 161 | 1 | C | 28.60% | 20.6 (SD =1.95) |
| Lieberman2005 | *Journal of health psychology* | Mixed | L | 114 | 1 | C | 0.00% | 46.2 (SD = 8.1) |
| March2018 | *Journal of Medical Internet Research* | Australia | C | 308 | 1 | C | 17.50% | 34.26 (SD = 11.23) |
| Marinova2022 | *Journal of Affective Disorders* | UK | L | 606 | 1 | C | 21.30% | 17.3 (0.71) |
| McKechnie2014 | *Journal of Medical Internet Research* | United Kingdom | L | 119 | 1 | C | 18.00% | 56 (SD = 11.29) |
| McNair2016 | *BMC Psychiatry* | Australia | C | 1628 | 1 | C | 0.00% | 34.09 |
| Meng2015 | *Psychiatry Research* | USA | C | 5645 | 0 | R | n/a | n/a |
| Merchant2022 | *Psychiatric Quarterly* | USA | C | 369 | 1 | C | 23.00% | 49 (15.6) |
| Millard2002 | *Disease Management & Health Outcomes* | USA | C | 10069 | 1 | R | 42.00% | 43 |
| Mo2013 | *Patient Education and Counseling* | Mixed | C | 340 | 1 | C | 83.70% | 47.81 (SD = 10.57) |
| Naslund2019 | *Early intervention in psychiatry* | Mixed (English speaking countries) | C | 135 | 1 | C | 31.90% | n/a |
| Nimrod2012a | *International Journal of Communication (19328036)* | Mixed | C | 558 | 1 | C | 30.00% | 36.1 (SD = 12.6) |
| Nimrod2012b | *Cyberpsychology* | Mixed | C | 793 | 1 | C | 30.00% | 36.05 (SD = 12.6) |
| Nimrod2013 | *Research on Cyberspace,* | Mixed | C | 793 | 1 | C | 30.00% | 36.05 (SD = 12.6) |
| Oh2017 | *Cin-Computers Informatics Nursing* | USA | C | 2351 | 1 | C | 49.60% | n/a |
| Park2016 (study1) | *Journal of Affective Disorders* | USA | C | 61 | 1 | C | 42.40% | 19.95 (SD = 1.13) |
| Park2016(study2) | *Journal of Affective Disorders* | USA | C | 42 | 1 | C | 86.00% | 24.95 (SD =7.40) |
| Powell2003 | *BMC Psychiatry* | Mixed (European) | C | 2037 | 1 | C | 30.00% | 30.5 |
| Roystonn2020 | *JMIR mental health* | Singapore | C | 6110 | 1 | R | 40.60% | n/a |
| Simmons2015 | *Public Health Nursing* | USA | C | 1000 | 1 | R | 0.00% | 57.31 (SD =16.09) |
| Teaford2015 | *Journal of Obstetric, Gynecologic, & Neonatal Nursing* | Mixed | C | 469 | 1 | C | 0%+T36:W3V36:W36 | 29.3 (SD = 5.3) |
| Toscos2018 | *Telemedicine and e-Health* | USA | C | 662 | 1 | C | 31.87% | 20.91 (SD = 1.69) |
| Toscos2019 | *JMIR mental health* | USA | C | 2789 | 1 | R | 45.39% | 16.09 (SD = 1.20) |
| Trail2020 | *Journal of medical Internet research* | USA | L | 212 | 1 | C | 6.10% | n/a |
| Van Meter2019 | *Journal of affective disorders* | USA | C | 32 | 1 | C | 40.00% | 21 (SD=3.2) |
| Wagner2004 | *Preventing chronic disease* | USA | C | 1980 | 3 | R | 41.70% | 54 |
| Wright2013 | *Journal of health communication* | USA | C | 361 | 1 | C | 46.00% | 20.26 (SD = 2.72) |
| Yu2020 | *Journal of Medical Internet Research* | China | C | 400 | 1 | R | 50.00% | 46.87 (SD =10.99) |
| Note:  *C = cross-sectional, L = longitudinal ** 1 = online, 0 = not online, 2 = mixed, 3 = telephone  *** R = Random, C= Convenient | | | | | | | | |
