# Supplementary material for: The Empowering Role of Web-Based Help Seeking on Depressive Symptoms: Systematic Review and Meta-analysis
Source: J Med Internet Res. 2023 Feb 2;25:e36964. doi: 10.2196/36964 (PMC9936373; doi:10.2196/36964)
Supplement: Multimedia Appendix 4 [file jmir_v25i1e36964_app4.docx]

Multimedia Appendix 4: Summary of Variables in Included Articles

This is a Multimedia Appendix to a full manuscript published in the J Med Internet Res. For full copyright and citation information see <http://dx.doi.org/10.219/3694>

| Study ID | Instrumental factors | Psychol. factors | Device | Frequency of visits | Patterns | Empowerment | Service use change |
| --- | --- | --- | --- | --- | --- | --- | --- |
| Akhther2022 | ✓ |  | OSG | ✓ | ✓ | ✓ |  |
| Algtewi2017 |  | ✓ | Self-help/App |  |  |  |  |
| Batenburga2014a | ✓ | ✓ | OSG |  |  |  |  |
| Batenburgb2014b | ✓ | ✓ | Internet, App |  |  |  |  |
| Brailovskaia2016 | ✓ | ✓ | OSG | ✓ | ✓ |  |  |
| DeAndrea2013 |  | ✓ | OSG |  |  |  |  |
| Fonseca2016 |  | ✓ | Internet |  |  |  |  |
| Frison2015 |  | ✓ | OSG | ✓ | ✓ |  |  |
| Giallo2017 |  |  | OSG |  |  | ✓ |  |
| Gold2016 | ✓ |  | OSG | ✓ | ✓ | ✓ |  |
| Grist2018 | ✓ | ✓ | Internet |  |  |  |  |
| Han2020 | ✓ |  | OSG |  |  | ✓ |  |
| Higueras2021 | ✓ |  | Internet | ✓ |  | ✓ |  |
| Houston2002 |  | ✓ | OSG |  |  |  |  |
| Huber2018 |  |  | OSG | ✓ | ✓ |  |  |
| Klemm2002 |  |  | OSG | ✓ | ✓ |  |  |
| Kobori2021 |  |  | OSG | ✓ |  | ✓ |  |
| Kohle2018 |  | ✓ | OSG |  |  |  |  |
| Kramer2015 |  |  | OSG | ✓ | ✓ |  |  |
| Kumar2020 |  |  | OSG |  |  |  |  |
| Leech2020 |  | ✓ | Internet-OSG |  |  |  |  |
| Lieberman2005 |  |  | OSG |  | ✓ | ✓ |  |
| March2018 | ✓ | ✓ | OSG | ✓ |  | ✓ | ✓ |
| Marinova2022 |  |  | OSG | ✓ |  |  |  |
| McKechnie2014 |  |  | OSG |  |  | ✓ | ✓ |
| McNair2016 |  |  | OSG |  | ✓ |  |  |
| Meng2015 |  |  | OSG | ✓ |  | ✓ |  |
| Merchant2022 |  |  | OSG |  |  |  |  |
| Millard2002 |  |  | OSG |  |  |  |  |
| Mo2013 |  |  | OSG |  |  |  |  |
| Naslund2019 |  |  | OSG |  | ✓ |  |  |
| Nimrod2012a |  |  | OSG |  |  |  |  |
| Nimrod2012b | ✓ |  | OSG |  |  | ✓ | ✓ |
| Nimrod2013 | ✓ |  | OSG | ✓ | ✓ | ✓ |  |
| Oh2017 |  |  | OSG | ✓ |  |  |  |
| Park2016 (study1) | ✓ |  | OSG | ✓ | ✓ | ✓ |  |
| Park2016 (study2) |  |  | OSG |  | ✓ | ✓ |  |
| Powell2003 | ✓ |  | OSG | ✓ | ✓ | ✓ |  |
| Roystonn2020 |  |  | Internet |  |  |  |  |
| Simmons2015 |  |  | OSG |  | ✓ | ✓ |  |
| Teaford2015 | ✓ |  | OSG |  |  | ✓ | ✓ |
| Toscos2018 |  |  | OSG | ✓ |  | ✓ |  |
| Toscos2019 |  |  | OSG |  |  |  |  |
| Trail 2020 |  |  | Self -help, OSG |  |  |  |  |
| Van Meter2019 |  |  | Internet |  |  |  |  |
| Wagner2004 | ✓ |  | Internet | ✓ |  | ✓ | ✓ |
| Wright2013 |  |  | OSG | ✓ | ✓ | ✓ |  |
| Yu2020 |  | ✓ | Self-help/App |  |  | ✓ |  |
