# Supplementary material for: The Empowering Role of Web-Based Help Seeking on Depressive Symptoms: Systematic Review and Meta-analysis
Source: J Med Internet Res. 2023 Feb 2;25:e36964. doi: 10.2196/36964 (PMC9936373; doi:10.2196/36964)

Multimedia Appendix 6: Funnel plot

This is a Multimedia Appendix to a full manuscript published in the J Med Internet Res. For full copyright and citation information see <http://dx.doi.org/10.219/3694>

Funnel plot for the the meta-analysis on Online Support Groups use and depressive symptoms.

Egger’s test for funnel plot asymmetry were not significant (t = -1.04, df = 12, p = .3171), thus indicating the absence of a publication bias.


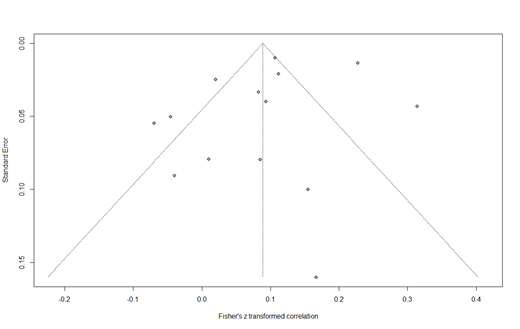

Supplement: Multimedia Appendix 6 [file jmir_v25i1e36964_app6.docx]
