# Supplementary material for: The Empowering Role of Web-Based Help Seeking on Depressive Symptoms: Systematic Review and Meta-analysis
Source: J Med Internet Res. 2023 Feb 2;25:e36964. doi: 10.2196/36964 (PMC9936373; doi:10.2196/36964)
Supplement: Multimedia Appendix 7 [file jmir_v25i1e36964_app7.docx]

Multimedia Appendix 7: Depression assessment

This is a Multimedia Appendix to a full manuscript published in the J Med Internet Res. For full copyright and citation information see <http://dx.doi.org/10.219/3694>

| **Type of measure** | | **Scale used** | **N** |
| --- | --- | --- | --- |
| **Self-report questionnaire for depressive symptoms** | Center for Epidemiologic Studies Depression Scale - 10 Items (CESD-10) | 3 |  |
|  | Center for Epidemiologic Studies Depression Scale - Revised (CESD-R) | 1 |  |
|  | Center for Epidemiologic Studies Depression Scale (CES-D; Radloff, 1997) | 6 |  |
|  | Center for Epidemiological Studies Depression Scale for Children (CES-DC) | 1 |  |
|  | Center for Epidemiologic Studies Depression Scale (Iowa short form) – 11 Items | 6 |  |
|  | Depression, Anxiety and Stress Scale - 21 Items (DASS-21) | 4 |  |
|  | Hospital Anxiety and Depression Scale | 4 |  |
|  | Major Depression Inventory | 1 |  |
|  | Patient Health Questionnaire - 9 Items (PHQ-9) | 5 |  |
|  | Patient Health Questionnaire - 2 Items (PHQ-2) | 2 |  |
|  | Patient Health Questionnaire - 4 Items (PHQ-4) | 3 |  |
|  | Patient Health Questionnaire - 8 Items (PHQ-8) | 2 |  |
|  | Edinburgh Postnatal Depression Scale (EPDS) | 3 |  |
|  | Ruminative response scale (RRS) | 1 |  |
| **Diagnostic interview** | World Health Organization Composite International Diagnostic Interview (CIDI) | 2 |  |
|  | Structured Clinical Interview for the DSM-IV, Patient Edition  (SCID-I/P) | 1 |  |
| **Audio computer-assisted self-interviewing (ACASI) format** | Major Depression (recent and past) | 1 |  |
| **Self-reported Diagnosis** |  | 6 |  |
