# Supplementary material for: The Empowering Role of Web-Based Help Seeking on Depressive Symptoms: Systematic Review and Meta-analysis
Source: J Med Internet Res. 2023 Feb 2;25:e36964. doi: 10.2196/36964 (PMC9936373; doi:10.2196/36964)
Supplement: Multimedia Appendix 8 [file jmir_v25i1e36964_app8.docx]

Multimedia Appendix 8: Empowerment Operationalization

This is a Multimedia Appendix to a full manuscript published in the J Med Internet Res. For full copyright and citation information see <http://dx.doi.org/10.219/3694>

| **Empowerment dimension** | **Article** | **Operationalization** | **Reference** |
| --- | --- | --- | --- |
| **Meaningfulness (n=11)** | **Brailovskaia2016** | Subjective experienced or anticipated support from the social network was measured with the one-dimensional German Questionnaire Social Support (F-SozU K-14). | Kliem, S., Mößle, T., Rehbein, F., Hellmann, D. F., Zenger, M., & Brähler, E. (2015). A brief form of the Perceived Social Support Questionnaire (F-SozU) was developed, validated, and standardized. Journal of clinical epidemiology, 68(5), 551-562. |
|  | **Han2020** | Level of help and support received in the community (Very much, Much, Less, Very little). 1 item | Ad hoc |
|  | **Huber2018** | Receiving useful information e.g. “I understand my illness better, I can accept my illness better; I can accept my illness better.” | Ad hoc |
|  | **Kramer2015** | If participants experienced benefit from the forum (yes, a little, no) | Ad hoc |
|  | **Millard2002** | Impact of health information that they received from the Internet on understanding health problems. | Ad hoc |
|  | **Mo2013** | Empowering processes scale e.g., “Receiving useful information, Information exchanged is of added value to the information I receive from my care providers”. | Mo, P. K., & Coulson, N. S. (2012). Developing a model for online support group use, empowering processes and psychosocial outcomes for individuals living with HIV/AIDS. Psychology & health, 27(4), 445-459. |
|  | **Nimrod2012a, Nimrod2012b, Nimrod2013** | Benefits of participation: list of 13 statements, which describe various benefits from participation in online depression communities. More specifically the following items: “I better understand my condition, I gain knowledge about various treatments”. | Ad hoc |
|  | **Park2016** | Perceptions of Facebook social support. Participants rated on a 4-point scale(1=strongly  disagree, 4=strongly agree) the extent to which they perceived themselves to have supportive Facebook friends (e.g., Among my  Facebook friends, there are people I can depend on to help me if I really need it.) | Ad hoc |
|  | **Powell2003** | Self-perceived effects of the communities. E.g., “I learned more about medication from using a community”. | Ah doc |
|  | **Wagner2004** | Whether using the Internet 1) improved understanding of the illness; 2) improved understanding of possible treatments for the illness. | Ah doc |
| **Self-efficacy (n=12)** | **Han2020** | Self-Efficacy Questionnaire for Chinese Family Caregivers (SEQCFC). | Zhang, S. Y., Edwards, H., Yates, P., Ruth, E., & Guo, Q. (2013). Preliminary reliability and validity testing of a Self-Efficacy Questionnaire for Chinese Family Caregivers. *Aging & mental health*, *17*(5), 630-637. |
|  | **Huber2018** | Motives for using peer-to-peer support and according experiences. More specifically the following item: “I feel better prepared for a doctor’s appointment”. | Ad hoc |
|  | **Nimrod2012a, Nimrod2012b, Nimrod2013** | Benefits of participation: list of 13 statements, which describe various benefits from participation in online depression communities. More specifically the following items: “My condition is under better control”; “I am more capable of dealing with daily tasks.” | Ad hoc |
|  | **Powell2003** | Self-perceived effects of the communities. More specifically the following item: “Gained ability to discuss subjects that they felt unable to discuss elsewhere.” | Ah doc |
|  | **Wagner2004** | Whether using the Internet improved the ability of respondent to manage the disease on his or her own | Ah doc |
| **Self-determination (n=1)** | **Huber2018** | Motives for using peer-to-peer support and according experiences e.g. “I feel more in control over what is happening to me” | Ad hoc |
| **Impact (n=1)** | **March2018** | The 18-item Multidimensional Health Locus of Control-Form C to assess the degree to which participants attributed their mental health to themselves or to external forces. | Wallston, K. A., Stein, M. J., & Smith, C. A. (1994). Form C of the MHLC scales: a condition-specific measure of locus of control. *Journal of personality assessment*, *63*(3), 534-553. |
